# Molecular Mimicry of the Rheumatoid Arthritis-Related Immunodominant T-Cell Epitope within Type II Collagen (CII260-270) by the Bacterial L-Asparaginase

**DOI:** 10.3390/ijms23169149

**Published:** 2022-08-15

**Authors:** Dzhemal Moten, Ivanka Teneva, Desislava Apostolova, Tsvetelina Batsalova, Balik Dzhambazov

**Affiliations:** Faculty of Biology, Plovdiv University “Paisii Hilendarski”, 24 Tsar Assen Street, 4000 Plovdiv, Bulgaria

**Keywords:** rheumatoid arthritis, HLA, T-cell receptor, type II collagen, L-asparaginase, molecular mimicry, autoimmunity

## Abstract

The etiology of most autoimmune diseases, including rheumatoid arthritis (RA), remains unclear. Both genetic and environmental factors are believed to be involved in pathogenesis. Molecular mimicry is considered one of the mechanisms for the occurrence of autoimmune diseases. The aim of the study was to determine whether the bacterial peptide L-ASNase67-81, which mimics the immunodominant T-cell epitope CII259-273, can induce T-cell reactivity in blood samples from RA patients and healthy subjects through molecular mimicry. Using bioinformatic molecular modeling methods, we first determined whether the L-ASNase67-81 peptide binds to the HLA-DRB1*04:01 molecule and whether the formed MHCII–peptide complex interacts with the corresponding T-cell receptor. To validate the obtained results, leukocytes isolated from early RA patients and healthy individuals were stimulated in vitro with L-ASNase67-81 and CII259-273 peptides as well as with bacterial L-asparaginase or human type II collagen (huCII). The activated T cells (CD4+CD154+) were analyzed by flow cytometry (FACS), and the levels of cytokines produced (IL-2, IL-17A/F, and IFN-γ) were measured by ELISA. Our in silico analyses showed that the bacterial peptide L-ASNase67-81 binds better to HLA-DRB1*04:01 compared to the immunodominant T-cell epitope CII259-273, mimicking its structure and localization in the binding groove of MHCII. Six contact points were involved in the molecular interaction of the peptide with the TCR. FACS data showed that after in vitro stimulation with the L-ASNase67-81 peptide, the percentage of activated T cells (CD154^+^CD4^+^) was significantly increased in both cell cultures isolated from ERA patients and those isolated from healthy individuals, as higher values were observed for the ERA group (9.92 ± 0.23 vs. 4.82 ± 0.22). Furthermore, the ELISA assays revealed that after stimulation with L-ASNase67-81, a significant increase in the production of the cytokines IL-2, IL-17A/F, and IFN-γ was detected in the group of ERA patients. Our data showed that the bacterial L-ASNase67-81 peptide can mimic the immunodominant T-cell epitope CII259-273 and activate HLA-DRB1*04:01-restricted T cells as well as induce cytokine production in cells isolated from ERA patients. These results are the first to demonstrate that a specific bacterial antigen could play a role in the pathogenesis of RA, mimicking the immunodominant T-cell epitope from type II collagen.

## 1. Introduction

Rheumatoid arthritis (RA) is one of the most prevalent autoimmune diseases, which is characterized by chronic inflammation and subsequent destruction of the affected joints. Although there are various hypotheses about the initiation of this autoimmune disease, the specific causes and exact mechanisms involved in the etiology of RA remain unclear. One of the most common hypotheses is that it involves infection with certain pathogenic microorganisms and subsequent molecular mimicry [1,2,3,4,5]. Molecular mimicry assumes sequence similarity between pathogenic and self-peptides that could provoke the cross-reactivity of the activated T or B cells to self-structures [6]. T lymphocytes (Th1 and Th17) are leading immune cells in the development of RA [6,7,8].

Although a number of bacteria are associated with the initiation of certain autoimmune diseases [2], no specific bacteria have yet been identified that are responsible for eliciting RA. Cusick et al. proposed that this is due to the involvement of multiple infections in the priming of the immune system and they gave examples of bacterial infections initiating or exacerbating autoimmune diseases [2]. In search of homology between bacterial proteins and RA-related T-cell epitopes using an immunoinformatics analysis, Repac et al. were able to retrieve the major RA-triggering candidates among which are *Proteus mirabilis*, *Escherichia coli*, *Mycobacterium tuberculosis*, *Klebsiella pneumoniae*, *Streptococcus pyogenes,* and *Prevotella* species [6]. A possible role of some oral and gut microbial species (*Porphyromonas gingivalis* and *Prevotella copri*) in RA pathogenicity has also been discussed [9,10]. During dysbiosis, these species can mediate the process of the citrullination of bacterial or human proteins triggering an immune response and production of anti-citrullinated antibodies that cross-react with human citrullinated peptides through molecular mimicry [9,10].

It is not necessary to look for a specific pathogenic microorganism that initiates the development of RA. It is possible that several pathogenic microorganisms contain the same proteins (antigens) that are homologous to the corresponding T-cell epitopes leading to the development of RA through molecular mimicry. In addition, these homologous proteins may not be part of the bacterial cell surface. They can also be intracellular, as ultimately after phagocytosis of pathogenic microorganisms to trigger an adaptive immune response, antigens are presented by antigen-presenting cells. Antigen-presenting cells (APCs), such as macrophages (Mφ) and dendritic cells (DCs), process the non-self (bacterial) antigens or self-antigens and present them through the major histocompatibility complex (MHC) and particularly the human leukocyte antigen (HLA) molecules to the T-cell receptors (TCRs) on T cells.

The association of HLA alleles with the increased risk for RA development has been intensively investigated [11,12,13]. HLA-DR alleles that have a particular sequence in the beta chain (HLA-DRB1) at positions 70–74 are known as “shared epitope alleles” (SE alleles) [11,14]. The presence of HLA-SE alleles increases the risk of developing RA and in most cases, this is associated with the production of anti-citrullinated protein antibodies (ACPA) in those RA patients (ACPA-positive RA patients) [9,10,11,15].

A major autoantigen candidate in RA pathogenesis is type II collagen (CII), the main protein component of articular cartilage. It has been shown that in both RA and collagen-induced arthritis (CIA) in mice, the autoreactive T cells predominantly recognize the same core of CII-encompassing residues 260–270 (CII260-270, IAGFKGEQGPK) [16,17,18,19,20,21]. This immunodominant T-cell epitope is restricted by the RA-associated HLA-DRB1*04 and HLA-DRB1*01 alleles [11,12,19,20,22]. Thus, to trigger autoreactive T cells via molecular mimicry, the foreign antigens have to be similar to the RA-related T-cell epitopes and presented within the right HLA molecules [6].

Bacterial L-asparaginase (L-ASNase, EC 3.5.1.1) shares amino acid sequence similarities (L-ASNase residues 68–78, IANVKGEQVVN) with CII260-270 [23,24]. This enzyme catalyzes the hydrolysis of L-asparagine to L-aspartate and ammonia and is absent in humans [24,25,26]. Many infectious pathogens suggested as major RA-triggering candidates (*Escherichia coli*, *Klebsiella pneumoniae*, representatives of the genera *Mycobacterium*, *Proteus*, *Streptococcus*) have been reported to produce L-asparaginase [25]. Therefore, the aim of this study was to clarify whether the peptide L-ASNase67-81, which is a part of bacterial L-asparaginase and mimics the RA-associated immunodominant T-cell epitope CII259-273, can induce T-cell reactivity in cells isolated from RA patients and healthy individuals by molecular mimicry.

By using binding prediction algorithms and molecular docking analyses we have demonstrated that bacterial peptide L-ASNase67-81 binds to the HLA-DRB1*04:01 molecule much better than the CII259-273 peptides (non-modified and galactosylated at position 264) representing the immunodominant T-cell epitope in RA. Further, molecular modeling also showed better interaction of the L-ASNase67-81/MHCII complex with the appropriate T-cell receptor (TCR) compared to the formed trimolecular complexes with the CII259-273 peptides. Functional tests performed in preselected rheumatoid arthritis patients and healthy subjects confirmed our hypothesis of the activation of the T-cell immune response by the bacterial peptide L-ASNase67-81 through molecular mimicry. Flow cytometry and ELISA analyses showed that the bacterial peptide was able to activate the T cells in the RA patients, increasing the expression of CD154 and the levels of produced IL-2, IL-17A/F, and IFN-γ cytokines.

## 2. Results

### 2.1. MHC Class II (MHCII) Binding Predictions and Molecular Docking

In order to confirm the possible antigenic mimicry in RA pathogenesis, we first evaluated the binding affinity of a synthetic peptide representing the sequence of the L-ASNase67-81 to HLA-DRB1 alleles in comparison with CII259-273, which is the major immunodominant T-cell epitope related to RA. The MHCII binding prediction results were retrieved from the Immune Epitope Database (IEDB) and are shown in Table 1.

The binding prediction algorithms showed an intermediate binding affinity with better values of the adjusted rank for the HLA-DRB1*04:01 allele (30% for CII259-273 and 28% for L-ASNase67-81). This allele was chosen for molecular modeling.

**Figure 1:**
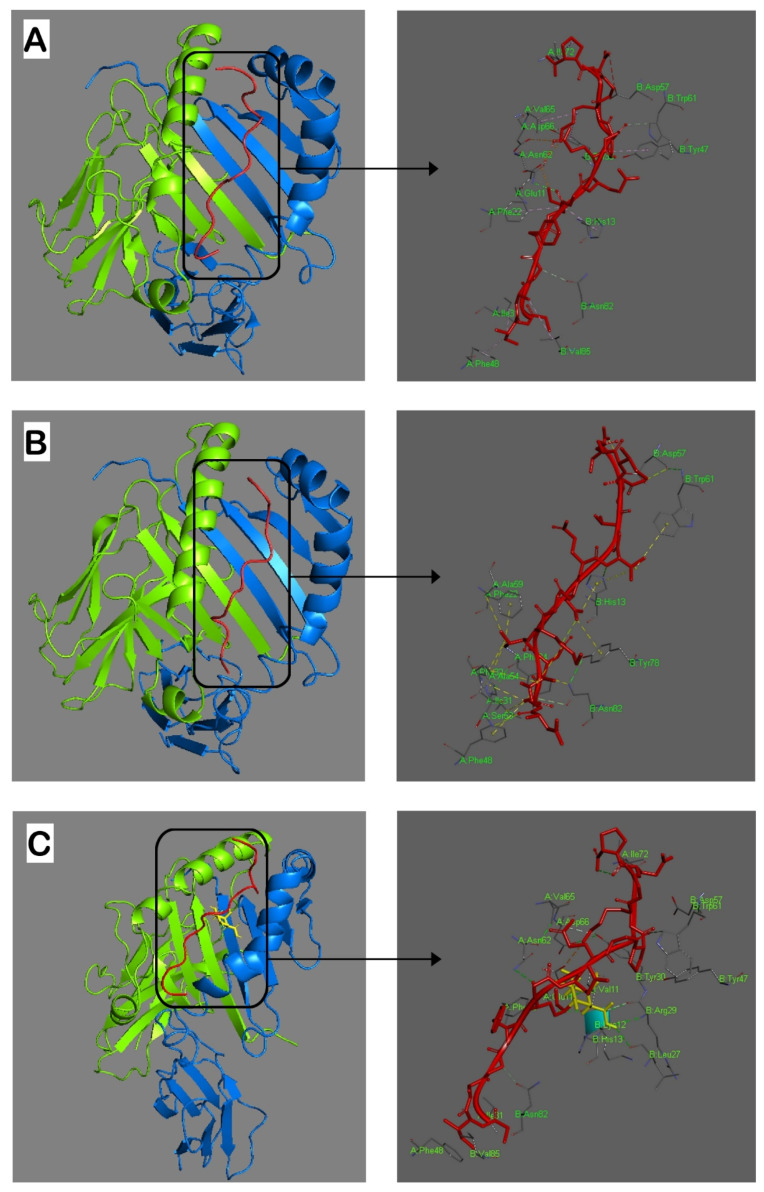
Graphical representation of interaction analyses between the HLA-DRB1*04:01 molecule and the studied peptides (red). (**A**): CII259-273; (**B**): ASNase67-81; (**C**): Gal264-CII259-273. Hydrogen bonds are indicated by dashed lines. Amino acid residues from both α and β chains important for interactions with the peptides are shown in bright green. Gal264 in CII259-273 peptide is yellow. Alpha and beta chains of the MHCII are bright green and blue, respectively. The docking was conducted by using ClusPro 2.2 and the results were visualized using Discovery Studio 2016 V16.1.0.

Next, we performed in silico molecular docking (Figure 1). Conformations with the lowest binding energy give the best docking results. The interaction between the immunodominant CII259-273 epitope and HLA-DRB1*04:01 was with a binding energy of −866.4 Kcal/mole (Figure 1A). A docking study of the L-ASNase67-81 peptide with the HLA-DRB1*04:01 molecule showed the lowest binding energy of –984.3 Kcal/mole (Figure 1B).

Since previous studies have reported that the autoreactive HLA-DRB1*04-restricted T cells in RA predominantly recognize CII259-273 when it is hydroxylated and galactosylated at K264 (GIAGFK[Gal-Hyl]GEQGPKGEP) [16,19,27], we also included in the docking analysis this modified epitope (Gal264-CII259-273). The energy-weighted score of interaction between Gal264-CII259-273 and HLA-DRB1*04:01 was −891.6 kcal/mole (Figure 1C). The docking structures showed that all investigated peptides were bound in the binding groove of HLA-DRB1*04:01 in a conserved linear conformation stabilized by different bonds (Figure 1).

The hydrogen bonds between the α or β chains of the HLA-DRB1*04:01 molecule on the one hand and the investigated peptides on the other, which are essential for the formation of the MHCII–peptide complex, are presented in Table 2. As can be seen from the structure models in Figure 1, the L-ASNase67-81 peptide mimics the conformation of the immunodominant CII259-273 epitope and even its binding energy is lower. This is a good sign that the L-ASNase67-81 peptide could be presented to the TCRs restricted by HLA-DRB1*04.

Thus, our analyses showed that the bacterial L-ASNase67-81 peptide binds to HLA-DRB1*04:01 much better than the immunodominant CII259-273 epitope and mimics its conformational structure.

### 2.2. Modeling of Interactions in the Trimolecular Complex HLA-DRB1*04:01—Peptide—TCR

**Figure 2:**
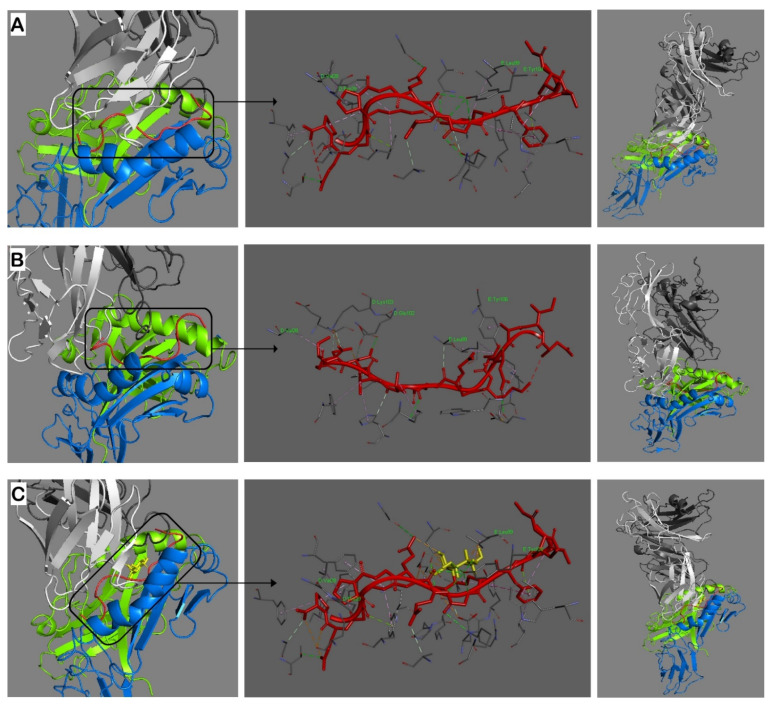
Molecular models of interaction in the trimolecular complex HLA-DRB1*04:01 (bright green/blue)—peptide (red)—α/β TCR (white/grey). (**A**): CII259-273 peptide; (**B**): ASNase67-81 peptide; (**C**): Gal264-CII259-273 peptide. Hydrogen bonds are indicated by dashed lines. Amino acid residues from both α and β chains of the TCR important for interactions with the peptides are shown in bright green. Gal264 in CII259-273 peptide is yellow. Alpha and beta chains of the MHCII are bright green and blue, respectively. Alpha and beta chains of the TCR are white and gray, respectively. The docking was conducted by using ClusPro 2.2 and the results were visualized using Discovery Studio 2016 V16.1.0.

Having established that the bacterial L-ASNase67-81 peptide is binding to the HLA-DRB1*04:01 groove with lower energy compared to the immunodominant T-cell epitope CII259-273, we next wanted to determine how this MHCII–peptide complex interacts with the TCR. To explore the capacity of possible molecular mimicry, the modeling was performed again for the three investigated peptides (Figure 2).

The molecular interaction of the L-ASNase67-81 peptide and the TCR occurs via three contact points with the α-chain (V28, E102, K103) and two contact points with the β-chain (L99, Y106) (Figure 2B, Table 3). In comparison, the immunodominant T-cell epitope CII259-273 interacts with V28 and P29 of the TCR α-chain and with L99 and Y104 of the TCR β-chain (Figure 2A, Table 3). Therefore, both peptides used the same V28 (TCR α-chain) and L99 (TCR β-chain) contact points but the L-ASNase67-81 peptide has one more contact with the α-chain (Table 3), suggesting better binding to the T-cell receptor and induction of the T-cell response.

Galactosylated CII259-273 peptide (Gal264-CII259-273) interacts with the TCR using the same TCR contact points (α-V28, α-P29, β-L99, β-Y104) as the non-modified CII259-273 epitope (Figure 2C) but in addition, also involved the galactose residue. Galactose binds only to L99 and Y104 of the TCR β-chain, with a total of five contact points (Table 3, values in bold).

Accordingly, the interaction between the complex L-ASNase67-81/MHCII and TCR is much more stable compared to that of the CII259-273/MHCII and TCR suggesting a possibility for molecular mimicry.

To further investigate the capacity of the bacterial L-ASNase67-81 peptide to activate T cells through molecular mimicry, we have performed functional validation tests using blood samples from early RA (ERA) patients and healthy volunteers.

### 2.3. Baseline Demographic and Disease Characteristics of Selected Early RA (ERA) Patients and Healthy Subjects

In our study, we examined a small cohort of ERA patients and healthy individuals that met predetermined criteria (see Materials and Methods). Twelve women with ERA and eleven healthy women (controls) aged 45 to 60 years, all of whom were non-smokers and had at least one copy of the HLA-DRB1*04:01 allele, were recruited for the study. Most of the patients had available data related to the selection criteria.

Only two patients were not tested for ACPA and their samples were screened together with those of the healthy subjects. The demographic and disease characteristics are shown in Table 4.

In addition, all recruited ERA patients were (1) diagnosed within 12 months, (2) ACPA-positive, (3) fulfilled the ACR/EULAR 2010 criteria, and (4) treated with methotrexate. Four of the ERA patients (33.33%) received concomitant therapy of prednisolone. The DAS28 values varied from 3.57 to 5.26 (mean 4.35 ± 0.16). Ten patients had moderate disease (DAS values were in the range between 3.57 and 4.87) and two patients were with high disease activity (DAS = 5.21 and DAS = 5.26, respectively).

Interestingly, two of the healthy women had increased levels of ACPA (88 U/mL and 96.4 U/mL, respectively) and rheumatoid factor (RF, 80 U/mL and 62 U/mL, respectively), but without any clinical signs of RA or other inflammatory/autoimmune diseases. Since the measured values were not high (compared to the ERA patients) these subjects were not excluded from the study.

### 2.4. Functional Validation of T-Cell Reactivity

**Figure 3:**
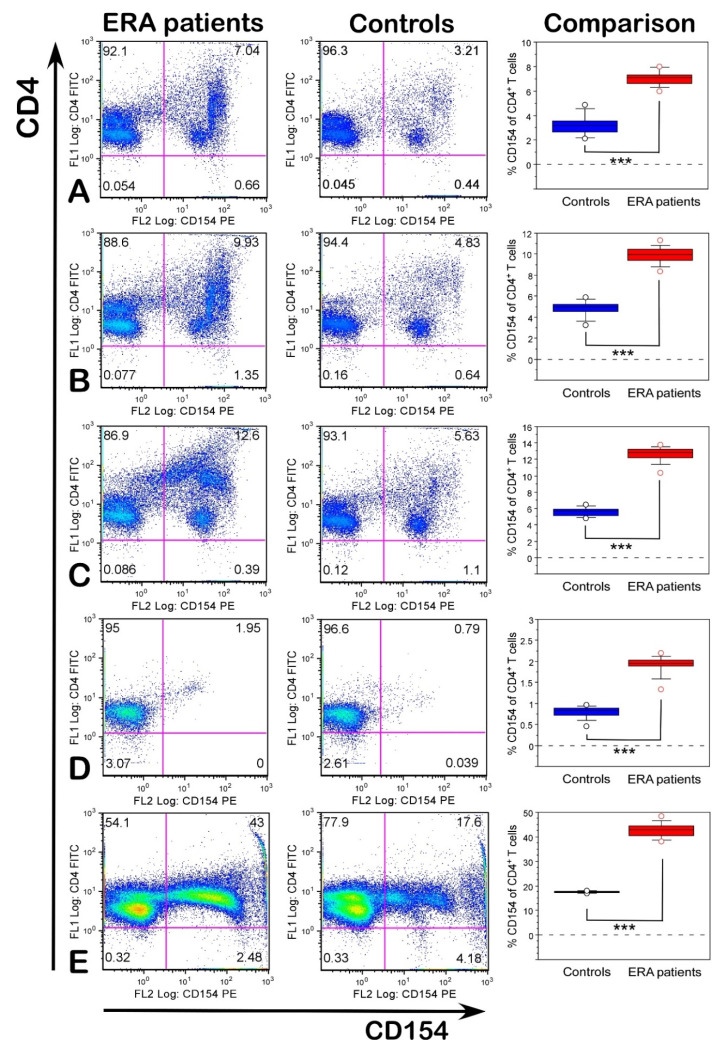
Expression levels of CD154 (% measured by flow cytometry) on CD4^+^ T cells in ERA patients and healthy individuals after in vitro stimulation with CII259-273 (**A**), L-ASNase67-81 (**B**), Gal264-CII259-273 (**C**), without stimulation (**D**), and with PHA-L (**E**). In the last column, (comparison) data are presented as means ± SE. *** *p* ≤ 0.001. Flow cytometry analyses were performed using a Cytomics FC500 flow cytometer.

To check whether the investigated antigens were functional and could activate T cells, isolated leukocytes from ERA patients and healthy subjects were stimulated in vitro with the peptides CII259-273, L-ASNase67-81, and Gal264-CII259-273, as well as with denatured huCII and bacterial L-ASNase. Since previous studies showed that activated antigen-specific CD4^+^ T cells can be detected by the upregulation of CD154 expression and this marker is associated with RA activity [19,28,29], we evaluated the expression of CD154 after stimulation with different antigens. As shown in Figure 3, both the ERA patients and healthy individuals responded to stimuli differently, as the ERA samples displayed a stronger T-cell response than those of the healthy subjects (*p* < 0.001).

Our analyses revealed that the percentage of CD154 on gated CD4+ T cells was highest in the samples from the ERA patients stimulated with Gal264-CII259-273 (12.62 ± 0.27, Figure 3C) followed by L-ASNase67-81 (9.92 ± 0.23, Figure 3B) and CII259-273 (7.04 ± 0.17, Figure 3A). In the healthy controls, these values were 5.63 ± 0.16, 4.82 ± 0.22, and 3.21 ± 0.25, respectively (Figure 3). Data showed that antigen-presenting cells (APC) carrying HLA-DRB1*04 alleles could present the peptide L-ASNase67-81 (that is a part of the bacterial L-asparaginase and mimics the immunodominant T-cell epitope CII259-273) and activate T cells. The expression levels of CD154 in the ERA patients were significantly higher than in the healthy subjects even when the cells were not stimulated (Figure 3D). This could be explained by the fact that the ERA patients already had activated T cells. The same tendency could also be seen in the positive control (cells stimulated with PHA-L), where the percentage of CD154^+^CD4^+^ T cells was significantly higher in the ERA patients compared to the controls (42.74 ± 0.84 vs. 17.49 ± 0.12, Figure 3E).

**Figure 4:**
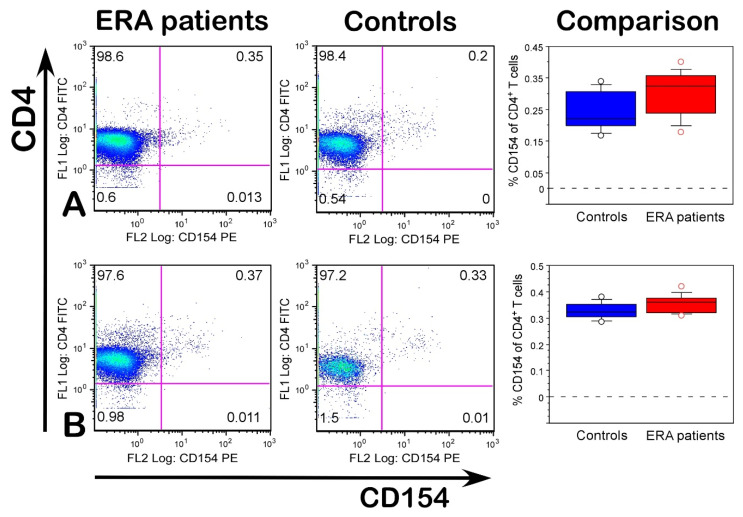
Percentage of CD154^+^CD4^+^ T cells in ERA patients and healthy individuals after in vitro stimulation with huCII (**A**) and bacterial L-asparaginase (**B**). In the last column, (comparison) data are presented as means ± SE. Flow cytometry analyses were performed by using a Cytomics FC500 flow cytometer.

The expression of CD154 had no significant difference between the ERA patients and healthy individuals after stimulation with denatured huCII and bacterial L-asparaginase (Figure 4). Furthermore, the percentage of CD154^+^CD4^+^ T cells even decreased compared with the baseline expression in non-stimulated cells (Figure 3D).

**Figure 5:**
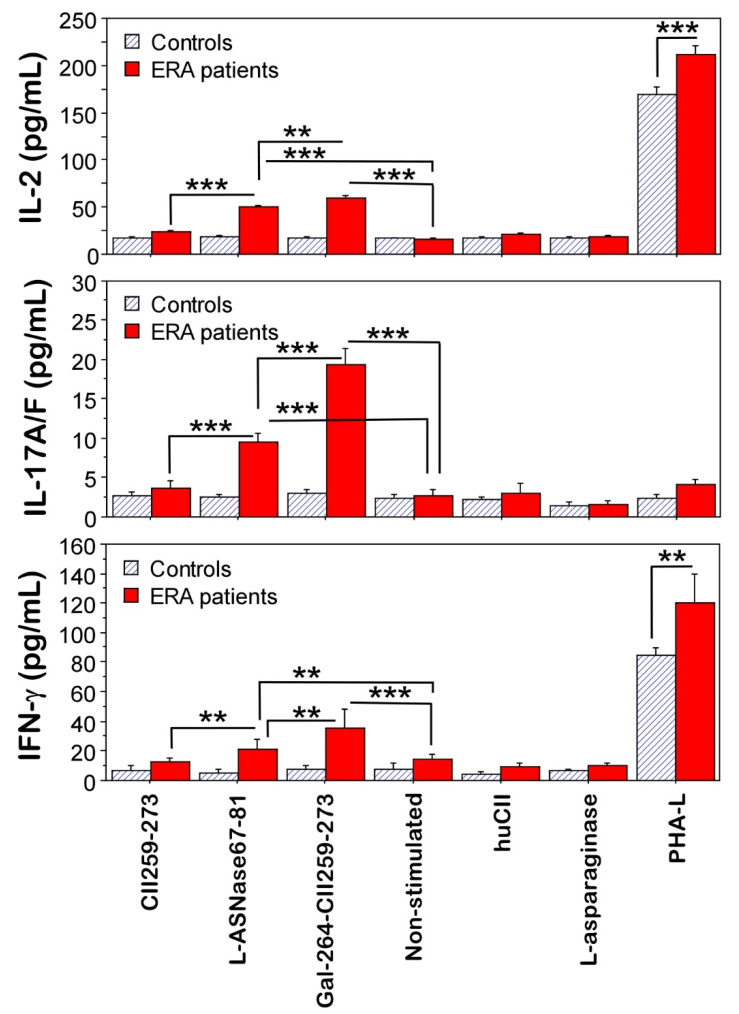
ELISA data for cytokine production by cultured leukocytes from ERA patients and healthy individuals (controls) after in vitro stimulation with different antigens for 96 h. Results are presented as means ± SE. ** *p* < 0.01, *** *p* < 0.001, as determined by Mann–Whitney U test.

Further, supernatants from the in vitro-stimulated cultures were investigated for the production of interleukin-2 (IL-2), interleukin-17 (IL-17A/F), and interferon-gamma (IFN-γ) (Figure 5). The overall secretion of cytokines was higher in the cultures of the ERA patients compared to the healthy controls. This is in agreement with the detected activated T cells (CD154^+^CD4^+^, Figure 3). Cells from the ERA patients stimulated with Gal264-CII259-273 and L-ASNase67-81 peptides produced significantly more IL-2 (59.5 ± 2.1 pg/mL and 49.9 ± 1.7 pg/mL, respectively), IL17A/F (19.3 ± 2.2 pg/mL and 9.6 ± 1.0 pg/mL, respectively), and IFN-γ (35.5 ± 8.6 pg/mL and 21.5 ± 4.4 pg/mL, respectively) than non-stimulated cells (15.8 ± 1.2 pg/mL, 2.7 ±7 pg/mL, and 14 ± 3.8 pg/mL) (Figure 5). Moreover, the bacterial L-ASNase67-81 peptide could induce the secretion of the cytokines IL-2, IL-17A/F, and IFN-γ from the activated T cells in the samples from the ERA patients. As can be seen in Figure 5, the highest production of cytokines was observed after stimulation of the cells from the ERA patients with the modified immunodominant T-cell epitope Gal264-CII259-273. The levels of IL-2 and IL17A/F were also increased in the ERA culture supernatants after stimulation with non-modified CII259-273 peptides compared to those without stimulation but did not reach statistical significance (Figure 5). No differences in cytokine concentrations were observed in cultures stimulated with huCII or L-asparaginase. The activated T cells from the antigen used as the positive control (PHA-L) secreted IL-2 and IFN-γ (but not IL-17A/F) in both groups (ERA patients and healthy controls) and again, the concentration of these cytokines was higher in the ERA group (Figure 5).

Therefore, our validation analyses showed that the investigated bacterial L-ASNase67-81 peptide is functional and able to activate HLA-DRB1*04:01-restricted T cells, inducing the expression of CD154 and production of cytokines.

## 3. Discussion

Usually, the contribution of microorganisms to the pathogenesis of RA is explained either by the local inflammation of mucosal surfaces or citrullination of bacterial and human proteins [9,10]. In the present study, we focused our investigations on molecular mimicry. We identified a specific bacterial epitope (L-ASNase67-81, part of the N-terminal domain of the bacterial L-asparaginase) that mimics the related RA-immunodominant T-cell epitope within type II collagen (CII259-273).

In order to reduce the influence of the genetic factor (MHCII) and smoking as an environmental factor, the present study selected participants who were non-smokers and expressed HLA-DRB1*04:01. To be recognized from TCRs and activate T cells, the mimicking foreign epitope has to be presented by certain HLA molecules on antigen-presenting cells. Here, we demonstrated that bacterial L-ASNase67-81 binds to HLA-DRB1*04:01 and even further, that this peptide can activate T cells in cell cultures by increasing the expression of CD154 and production of cytokines (IL-2, IL17A/F, IFN-γ).

Comparative molecular modeling showed similar structure conformations and localization in the binding groove of HLA-DRB1*04:01 for the three investigated peptides (L-ASNase67-81, CII259-273, and Gal264-CII259-273). The similarity is probably due to the same contact points (three on the α-chain and three on the β-chain of the MHCII) that are used in the studied peptides. In addition to these common contact points, as can be seen in Figure 1 and Table 2, the peptides stabilize their interaction with MHCII using other hydrogen bonds. Our data for the binding of CII259-273 and Gal264-CII259-273 peptides to HLA-DRB1*04:01 differ from previously described models [30,31,32]. The reason is that we selected the balanced models with the lowest binding energies from all the generated models without making any adjustments.

The molecular interactions of the formed MHCII–peptide complexes with the TCR revealed that the L-ASNase67-81 peptide has six contact points with the TCR (three for the α-chain and three for the β-chain), whereas the immunodominant CII259-273 epitope has five contact points (two for the α-chain and three for the β-chain). The modified epitope Gal264-CII259-273 uses the same contact points as the non-modified but in addition, the galactose at position 264 stabilizes the interaction with the TCR by using five contact points directed to the β-chain of the TCR. Interestingly, Ge et al. reported that the peptide Gal264-CII259-273 interacts with the β-chain of the TCR only with one salt bridge (K270-D29), and with one salt bridge (K264-D94) and one hydrogen bond (K266-N97) with the α-chain of the TCR [31]. Furthermore, the galactose residue at K264 is not involved in side chain interactions with the TCR and they showed only one hydrogen bond interacting with the α-chain of the TCR (Gal-L96) [31]. This is strange because other functional studies (including the present study) demonstrated that T cells predominantly recognize Gal264-CII259-273 in comparison with the non-modified epitope CII259-273 [16,19,27]. Our data showed that Gal264 interacts with L99 and Y104 of the β-chain of the TCR using five contact points (Table 3).

Importantly, our functional studies on the blood samples from the ERA patients and healthy individuals supported our hypothesis and showed that bacterial L-ASNase67-81 could activate T cells by molecular mimicry inducing the expression of CD154 in both the ERA and control groups and cytokine production in the ERA group. The highest expression levels of CD154 were observed in the cultures from the ERA patients after stimulation with modified Gal264-CII259-273 epitope (sixfold higher than non-stimulated cells) followed by those stimulated with bacterial L-ASNase67-81 peptide (fivefold higher than non-stimulated cells). The expression levels of CD154 in the cultures from the ERA patients stimulated with non-modified CII259-273 increased 3.5-fold compared to the non-stimulated cells. Notably, in the cultures of the healthy subjects, the levels of CD154 were also increased after stimulation with these three peptides, keeping the same tendencies and similar fold increases. Generally, the expression levels of CD154 were higher in the ERA patients than in the control group. Our results are consistent with previous studies that also reported the higher expression of CD154 in RA patients compared to healthy controls [29,33,34]. This was explained by the Ca^2+^ influx [35]. The decreased levels of CD154 in the cultures from both the ERA patients and control subjects after stimulation with denatured proteins (huCII or L-asparaginase) could be explained either by the need for protein processing or the increased number of activated macrophages followed by the competitive binding of Mac-1 to CD154 [36].

Moreover, CD154 expression is reflected in cytokine production. Increased levels of the cytokines IL-2, IL17A/F, and IFN-γ were determined only in the ERA cultures after stimulation with the investigated peptides. The concentrations of the cytokines followed the expression levels of CD154. No cytokine secretion was detected in the cultures from the healthy individuals, although activated T cells (CD4^+^CD154^+^) were identified. Similar results were reported by Snir et al. investigating T-cell reactivity to the citrullinated peptide vimentin residues 59–78 in RA patients [34]. The authors identified IFN-γ as a key cytokine in anticitrullinated immunity [34]. The lack of a T-cell response in cell cultures from healthy individuals after stimulation with the studied peptides can be explained by the lack of antigen-specific T cells. Therefore, cytokine production cannot be expected. This is also supported by the results obtained after stimulating the cell cultures with PHA-L, the antigen used as the positive control. PHA-L induced the production of IL-2 and IFN-γ but not IL-17A/F. The cytokine IL17A/F was only detected in the cultures of patients with ERA (where Th17 cells are present) and when they were stimulated with the correct antigen. Th17 cells are mediators of inflammation and autoimmunity and secrete IL-17, IL-21, and IL-22 [37]. In addition, Th17 cells cross-react with B cells, implicating them in the further immunopathogenesis of RA [9,38]. In future studies, it would also be interesting to investigate the involvement of B cells. In this study, this was impossible because, after a long culture, the B cells are dead.

To our knowledge, this is the first study to demonstrate that a specific foreign antigen (bacterial L-ASNase67-81) can activate T cells isolated from ERA patients through molecular mimicry. Our findings confirm the hypothesis that antigens from different pathogenic microorganisms can cross-react with certain TCRs triggering an autoimmune T-cell response in susceptible individuals.

## 4. Materials and Methods

### 4.1. Antigens

Human collagen type II (huCII), L-asparaginase (L-ASNase) from *Escherichia coli,* and phytohemagglutinin-L (PHA-L) from *Phaseolus vulgaris* were purchased from Merck KGaA (Darmstadt, Germany). Synthetic peptides CII259-273 (GIAGFKGEQGPKGEP, representing the native non-modified T-cell epitope within collagen type II) and L-ASNase67-81 (DIANVKGEQVVNIGS) were produced by Schafer-N (Copenhagen, Denmark). Modified at position K264, CII259-273 peptide (Gal264-CII259-273, GIAGFK[Gal-Hyl]GEQGPKGEP) by linkage of β-D-galactopyranosyl residue to 5-hydroxy-L-lysine was produced by Syngene (Bangalore, India).

### 4.2. MHCII Binding Predictions and Molecular Docking

The MHCII binding predictions were made on 5 July 2022 using binding prediction algorithms in the Immune Epitope Database (IEDB, https://www.iedb.org/) and the analysis resource Consensus tool [39,40].

In order to estimate the binding affinities between the HLA-DRB1*04:01 allele and the peptides CII259-273, L-ASNase67-81, and Gal264-CII259-273, in silico molecular docking was used. The three-dimensional structure of the MHC class II allele DRB1*04:01 (PDB ID: 7NZE) was retrieved from the RCSB Protein Data Bank (http://www.rcsb.org/pdb/home/home.do) as a PDB file. Since the retrieved crystal structure was in the complex form of protein and ligand, Discovery Studio 2016 V16.1.0 was used to separate the protein and ligand from the complex structure [41]. All water molecules and heteroatoms in the retrieved target file 7NZE were then removed. For docking studies, the non-modified CII259-273 (GIAGFKGEQGPKGEP) and L-ASNase67-81 (DIANVKGEQVVNIGS) peptides were subjected to a PEP-FOLD server for 3D structure formation [42]. It created 5 models of each peptide and the best model was chosen. The modified Gal264-CII259-273 peptide (GIAGFK[Gal-Hyl]GEQGPKGEP) was composed by Discovery Studio 2016 V16.1.0 [41]. Docking studies were performed with ClusPro 2.2 web server (https://cluspro.bu.edu/) [43]. Ten confirmations for each of the studied epitopes were produced. The best-docked complex was selected based on the lowest binding energy.

To evaluate the interactions between the MHCII–peptide complexes and the TCR, in silico molecular docking was performed using the template of a published TCR cocrystallized with an influenza peptide-containing DRB1*04:01 molecule (1J8H) [44]. The file was modified with Discovery studio v16.1.0 and energy minimized with UCSF Chimera [41,45].

### 4.3. Patients, Human Leucocyte Antigen (HLA), Genotyping, and Measurement of ACPA Levels and Rheumatoid Factor (RF)

To minimize the interpatient heterogeneity of the clinical phenotypes, we decided to use a small patient cohort with specifically defined parameters. Inclusion criteria were as follows: females between 45 and 60 years of age, disease symptoms are less than 12 months since diagnosis (early RA, ERA), fulfil the American College of Rheumatology (ACR)/European League Against Rheumatism (EULAR) 2010 criteria for the classification of RA [46], SE-positive (at least one copy of the HLA-DRB1*04:01 allele), ACPA-positive (>50 U/mL), presence of IgM rheumatoid factor (RF > 25 U/mL), and non-smokers. These baseline demographic and disease characteristics were collected from the medical files of the patients in collaboration with a rheumatologist. In addition, the patients had to be treated only with methotrexate (up to 20 mg weekly). A low corticosteroid dosage (≤5 mg/day) was permitted, but for up to three months. All 38 eligible patients were invited to participate in the study, but not all of them were included due to personal reasons related to travel or changing the therapy used. Healthy staff members of similar age from the Paisii Hilendarski University of Plovdiv served as the healthy controls. Inclusion criteria for the group of healthy controls were females between 45 and 60 years of age with no reported inflammatory joint disease, SE-positive (at least one copy of the HLA-DRB1*04:01 allele), and non-smokers. The final study involved 12 ERA patients and 11 healthy control individuals (after HLA genotyping of 86 healthy volunteers). Baseline data about the participants are given in Table 4. None of the participants received medications during the last 48 h before blood collection. Disease activity of the ERA patients was assessed by evaluating the Disease Activity Score (DAS28) calculated with the level of C-reactive protein (CRP) on the day of the blood sampling, quantified by a Latex agglutination slide test (Humatex, Wiesbaden, Germany). All participating individuals provided written informed consent according to the Declaration of Helsinki. Sample collection was approved by the Local Ethical Committee at the Paisii Hilendarski University of Plovdiv under No. 5/10.06.2020.

Genomic DNA for HLA genotyping of the healthy subjects was isolated from whole blood samples using QIAamp DNA Mini kits for genomic DNA purification (Qiagen GmbH, Hilden, Germany) according to the manufacturer’s instructions. After quantitation of DNA by NanoDrop 2000 UV-Vis spectrophotometer (Thermo Fisher Scientific, Wilmington, DE, USA), 100 ng DNA was used for the PCR amplification of exons 2-3 of the HLA-DRB1 gene (GeneQuery™ HLA-DRB1 PCR+Sanger SBT Typing Kit, ScienCell™ Research Laboratories, Carlsbad, CA, USA). Amplified PCR products were purified using a SpeeDNA PCR Purification Kit (ScienCell™ Research Laboratories, Carlsbad, CA, USA), and following the manufacturer’s instructions, the PCR products were sent for sequencing (Eurofins, Ebersberg, Germany). Four-digit HLA-DRB1 genotypes were determined using SOAPTyping software (https://github.com/BGI-flexlab/SOAPTyping).

The presence of anti-CCP3 antibodies in the sera of enrolled healthy subjects and ERA patients without such data was determined using the Quanta Lite CCP 3.1 IgG/IgA ELISA kit (INOVA Diagnostics Inc, San Diego, CA, USA). Rheumatoid factor detection was performed using a Roche/Hitachi Cobas C system (Roche Diagnostics International AG, Rotkreuz, Switzerland).

### 4.4. Functional Validation of T-Cell Reactivity

#### 4.4.1. Cell Isolation and Stimulation

Peripheral blood was collected into BD Vacutainer^®^ K2EDTA tubes (Becton, Dickinson and Company, Oakville, ON, Canada) in a clinical laboratory. Blood samples were centrifuged at 1500× *g* for 15 min at room temperature, and the sera were collected and stored at −80 °C until use for other measurements. Next, red blood cells were lysed with 0.84% NH_4_Cl buffer, and after washing twice with a sterile Dulbecco’s Phosphate-Buffered Saline (D-PBS, Gibco^®^, Life Technologies™, Paisley, Scotland, UK), the isolated leukocytes were resuspended in RPMI 1640 Complete Medium, with 2 mM L-Glut and 10% FBS, supplemented with antibiotic-antimycotic solution (all from Merck KGaA, Darmstadt, Germany). Cells were cultured at a concentration of 1 × 10^6^ cells/mL in 96-well plates (TPP, Trasadingen, Switzerland) at 37 °C, 5% CO_2_, 95% atmospheric air in RPMI 1640 Complete Medium, with 2 mM L-Glut and 10% FBS, supplemented with antibiotic-antimycotic solution (all from Merck KGaA, Darmstadt, Germany) for 96 h in presence/absence of the investigated peptides (CII259-273, L-ASNase67-81, Gal264-CII259-273) at a concentration of 10 µg/mL or denatured (incubated at 56 °C for 45 min) proteins (huCII, L-ASNase) at a concentration of 50 µg/mL. Cells, cultured under the same conditions in presence of 1 µg/mL PHA-L were used as the positive control. All in vitro stimulations have been performed in triplicate.

#### 4.4.2. Fluorescence-Activated Cell Sorting (FACS) Analysis

On day 5, cells were collected by centrifugation at 1500× *g* for 10 min, resuspended in FACS buffer (D-PBS, supplemented with 5% FBS and 0.05% NaN_3_), and stained for 20 min at 4 °C with fluorescently labeled monoclonal antibodies against the surface molecules CD4 and CD154 (anti-human CD4-FITC and anti-human CD154-PE, both from BioLegend^®^, San Diego, CA, USA). After washing with FACS buffer, the analysis was performed using a Cytomics FC500 flow cytometer (Beckman Coulter Inc., Life Sciences, Indianapolis, IN, USA).

#### 4.4.3. Cytokine Measurements in Supernatants by ELISA

Cytokine content (IL-2, IL-17, and IFN-γ) in the supernatants of stimulated/non-stimulated cells was measured by LEGEND MAX™ Human IL-2, Human IL-17A/F, and Human IFN-γ ELISA kits (BioLegend^®^, San Diego, CA, USA) according to the manufacturer’s instructions. Absorbance was measured at a 450 nm wavelength using a SpectraMax i3x Multi-Mode Microplate Reader (Molecular Devices LLC, San Jose, CA, USA). The sensitivity of the assay for IL-2, IL-17, and IFN-γ was 4 pg/mL, 2.58 pg/mL, and 5.6 pg/mL, respectively.

### 4.5. Statistics

Statistical analyses were performed using the StatView software (SAS Institute, Carry, NC, USA). The Mann–Whitney U test was used to compare the means of the two groups (ERA patients vs. healthy subjects). *p* values less than 0.05 were considered statistically significant. Experimental data are presented as mean ± standard error (SE).

## 5. Conclusions

In this study, we investigated the possible contribution to RA pathogenesis of a specific bacterial peptide (L-ASNase67-81) that has a similar amino acid sequence to the RA-associated immunodominant T-cell epitope CII259-273. The performed comparative molecular modeling revealed that the bacterial peptide had a better binding affinity to MHCII and a better interaction with the TCR compared to the immunodominant T-cell epitope. The functional tests showed that L-ASNase67-81 can activate HLA-DRB1*04:01-restricted T cells isolated from ERA patients through molecular mimicry inducing the expression of CD154 and cytokine production (IL-2, IL17A/F, and IFN-γ). Our data demonstrate that antigens from different pathogenic microorganisms could play a role in the pathogenesis of RA, triggering an autoimmune T-cell response in susceptible individuals. Therefore, these results can be used in clinical practice, especially in the initial determination of the treatment regimen of patients with early RA.

## Figures and Tables

**Table 1:** Binding properties of the peptides CII259-273 and L-ASNase67-81 to the RA-associated HLA-DRB1 alleles.

HLA-DRB1 Allele	Peptide Sequence	Method ^#^	Prediction Score (1—log50K)	Affinity IC_50_ in nM)	Adjusted Rank (%)
HLA-DRB1*01:01	CII259-273	Consensus	0.5438	139.2	35.00
	L-ASNase67-81	Consensus	0.6301	54.7	48.00
HLA-DRB1*04:01	CII259-273	Consensus	0.5352	152.7	30.00
	L-ASNase67-81	Consensus	0.3810	810.7	28.00

^#^ The consensus method considers a combination of any three of the four methods (combining NN-align, SMM-align, CombLib, and Sturniolo), if available, where Sturniolo is a final choice.

**Table 2:** Hydrogen bonds between the HLA-DRB1*04:01 molecule and the peptides CII259-273, L-ASNase67-81, and Gal264-CII259-273.

HLA-DRB1*04:01	CII259-273	L-ASNase67-81	Gal264-CII259-273
***α chain***			
Glu(E)11	Lys(K)264—3.18 Å	—	Lys(K)264—3.56 Å
Phe(F)22	Lys(K)264—5.27 Å	Val(V)71—3.99 Å	Lys(K)264—5.27 Å
Ile(I)31	Ile(I)260—4.45 Å	Ala(A)69—4.25 Å	Ile(I)260—4.45 Å
Phe(F)32	—	Ala(A)69—4.12 Å	—
Phe(F)48	Ile(I)260—4.58 Å	Ala(A)69—4.53 Å	Ile(I)260—4.58 Å
Ala(A)54	—	Ile(I)68—4.87 Å	—
Ala(A)59	—	Val(V)71—5.04 Å	—
Asn(N)62	Lys(K)264—2.68 Å	—	Lys(K)264—2.68 Å
Val(V)65	—	—	Lys(K)270—5.04 Å
Asp(D)66	Lys(K)270—5.10 Å	—	—
Ile(I)72	Pro(P)273—5.04 Å	—	Pro(P)273—5.04 Å
*****β chain*****			
His(H)13	Lys(K)264—4.37 Å	Val(V)76—5.33 Å	—
Leu(L)27	—	—	Gal-Hyl264—2.90 Å
Tyr(Y)30	Lys(K)270—3.26 Å	—	Lys(K)270—5.12 Å
Tyr(Y)47	—	—	Pro(P)269—4.99 Å
Asp(D)57	—	Asn(N)78—3.00 Å	—
Trp(W)61	Gly(G)268—2.92 Å	Val(V)76—5.48 Å	Gly(G)268—2.92 Å
Tyr(Y)78	—	Glu(E)74—4.33 Å	—
Asn(N)82	Gly(G)262—3.18 Å	Ala(A)69—3.40 Å	Gly(G)262—3.18 Å
Val(V)85	Ala(A)261—4.82 Å	—	—

**Table 3:** Important hydrogen bonds between a human α/β T-cell receptor and the peptides CII259-273, L-ASNase67-81, and Gal264-CII259-273.

Human α/β T-Cell Receptor	CII259-273	L-ASNase67-81	Gal264-CII259-273
***α chain***			
Val(V)28	Pro(P)269—4.37 Å	Ile(I)79—4.25 Å	Pro(P)269—4.37 Å
Pro(P)29	Pro(P)269—5.42 Å	—	Pro(P)269—4.37 Å
Glu(E)102	—	Asp(D)78—3.09 Å	—
Lys(K)103	—	Ser(S)81—2.73 Å	—
***β chain***			
Leu(L)99	Lys(K)264—5.06 Å	Glu(E)74—3.29 Å	Lys(K)264—5.06 Å
			**Gal:C1(2.25Å); Gal:C2(2.27Å); Gal:O2(2.21Å)**
Tyr(Y)104	Gly(G)265—2.90 Å	—	Gly(G)265—2.90 Å; Glu(E)266—2.82 Å;
	Glu(E)266—2.82 Å	Ala(A)69—5.11 Å	**Gal:HO2(2.41 Å); Gal:O3(2.74 Å)**
Tyr(Y)106	—	Asn(N)70—2.66 Å	—

**Table 4:** Baseline demographic and disease characteristics * of ERA patients and healthy controls.

Characteristics	ERA Patients	Healthy Controls
Number of subjects	12	11
Sex (female, %)	100	100
Age (years)	53 ± 1 (45–60)	52 ± 2 (45–60)
HLA-DRB1*04:01 (%)	100	100
Non-smokers (%)	100	100
Disease duration (months)	9 ± 1	—
Anti-CCP3 level (U/mL)	205.2 ± 26.0	31.5 ± 10.0
RF (U/mL)	171.8 ± 16.6	21.2 ± 7.6
DAS28 (CRP)	4.35 ± 0.16	—
CRP (mg/L)	22.5 ± 2.0	—
Methotrexate, MTX (%)	100	—
MTX dosage (mg/week)	10.8 ± 0.9	—
Prednisolone (%)	33.33	—
Prednisolone dosage (mg/day)	5.0 ± 0.0	—

* Data are presented as mean values ± SE. SE: standard error.

## Data Availability

Data are contained within the article or available from the corresponding author upon request.
